# A neuropathologic feature of brain aging: multi-lumen vascular profiles

**DOI:** 10.1186/s40478-023-01638-2

**Published:** 2023-08-28

**Authors:** Eseosa T. Ighodaro, Ryan K. Shahidehpour, Adam D. Bachstetter, Erin L. Abner, Ruth S. Nelson, David W. Fardo, Andy Y. Shih, Roger I. Grant, Janna H. Neltner, Frederick A. Schmitt, Gregory A. Jicha, Richard J. Kryscio, Donna M. Wilcock, Linda J. Van Eldik, Peter T. Nelson

**Affiliations:** 1 Department of Neurology, Emory University, Atlanta, GA USA; 2 Sanders-Brown Center On Aging, University of Kentucky, Rm 575 Lee Todd Bldg, 789 S. Limestone Ave, Lexington, KY 40536 USA; 3 Department of Neuroscience, University of Kentucky, Lexington, KY 40536 USA; 4 Spinal Cord and Brain Injury Research Center, University of Kentucky, Lexington, KY 40536 USA; 5 Department of Epidemiology and Environmental Health, University of Kentucky, Lexington, KY 40536 USA; 6 Department of Pediatrics, Center for Developmental Biology and Regenerative Medicine, Seattle Children’s Research Institute, University of Washington, Seattle, WA 98101 USA; 7 Department of Neurosciences and Center for Biomedical Imaging, Medical University of South Carolina, Charleston, SC 29425 USA; 8 Department of Pathology and Laboratory Medicine, Division of Neuropathology, University of Kentucky, Lexington, KY 40536 USA; 9 Department of Neurology, University of Kentucky, Lexington, KY 40536 USA; 10 Department of Statistics, University of Kentucky, Lexington, KY 40536 USA; 11 Department of Biostatistics, University of Kentucky, Lexington, KY 40536 USA; 12 Emory University, Atlanta, GA USA

**Keywords:** VCID, Small vessel disease, SVD, TBI, VTE, Senescence, Endothelial

## Abstract

Cerebrovascular pathologies other than frank infarctions are commonly seen in aged brains. Here, we focus on multi-lumen vascular profiles (MVPs), which are characterized by multiple vessel lumens enclosed in a single vascular channel. Little information exists on the prevalence, risk factors, and co-pathologies of MVPs. Therefore, we used samples and data from the University of Kentucky Alzheimer’s Disease Research Center (*n* = 91), the University of Kentucky Pathology Department (*n* = 31), and the University of Pittsburgh Pathology Department (*n* = 4) to study MVPs. Age at death was correlated with MVP density in the frontal neocortex, Brodmann Area 9 (*r* = 0.51; *p* < 0.0001). Exploratory analyses were performed to evaluate the association between conventional vascular risk factors (e.g., hypertension, diabetes), cardiovascular diseases (e.g., heart attack, arrhythmia), and cerebrovascular disease (e.g., stroke); the only nominal association with MVP density was a self-reported history of brain trauma (Prevalence Ratio = 2.1; 95 CI 1.1–3.9, before correcting for multiple comparisons). No specific associations were detected between neuropathological (e.g., brain arteriolosclerosis) or genetic (e.g., *APOE*) variables and MVP density. Using a tissue clearing method called SeeDB, we provide 3-dimensional images of MVPs in brain tissue. We conclude that MVPs are an age-related brain pathology and more work is required to identify their clinical-pathological correlation and associated risk factors.

**Supplementary Information:**

The online version contains supplementary material available at 10.1186/s40478-023-01638-2.

## Introduction

The human cerebral vasculature is responsible for numerous functions such as oxygen loading, nutrient transport, mediation of neuroinflammation, and removal of harmful materials [1–3]. With aging and cerebrovascular diseases, the structure of blood vessels can exhibit dramatic remodeling [1, 2, 4, 5]. Specific small vessel pathologic features include arteriolosclerosis, arteriovenous malformations, and downstream changes such as lacunar infarcts, leukoaraiosis, micro-infarcts, and hemorrhagic lesions [4, 6–9]. However, the histomorphologic features related to cerebrovascular malfunction are more heterogeneous than is widely appreciated, and here we focus on a subtype of vascular pathology about which there is relatively little information published.

Terms that have been applied in the literature to describe small blood vessels with multiple lumens enclosed in a single perivascular space include: vascular loops, vascular bundles, vascular convolutes, vascular spirals, vascular multiplications, raspberries, and vascular glomerular loop formations [10–14]. However, there is presently no rigorous definition for these vessels. Within this article, we use the term multi-lumen vascular profile (MVP) to describe a vascular profile consisting of ≥ 3 lumens enclosed in a perivascular space on a cross-sectional view. The pathophysiologic significance of MVPs is obscure. Previous researchers described MVP-like “convoluted vessels” in individuals with senile dementia [10, 13, 15, 16]. There are other reports of glomeruloid-like structures being seen in the brains of elderly individuals [11, 13, 17–19]. It has been suggested that MVPs arise due to hypoxic/ischemic changes in the brain [13, 14, 18, 19]. However, the vascular risk factors, vascular diseases, and co-pathologies have not been systematically investigated. Therefore, the purpose of this manuscript was to study this surprisingly common pathologic entity in human material. To investigate the frequency, risk factors, and co-pathologies of MVPs, we analyzed brains of individuals who came to autopsy at the University of Kentucky and the University of Pittsburgh.

## Methods

This study used human brain samples and data from the University of Kentucky Alzheimer’s Disease Research Center (UK-ADRC), the University of Kentucky Pathology Department (UKPD), and the University of Pittsburgh Pathology Department (UPPD). Patient recruitment, tissue and data collection in the UK-ADRC research study have been previously described including details related to institutional review board approval and patient consent [20, 21]. Human tissue samples from UKPD resulted from autopsies that were performed after obtaining informed consent using forms approved by the Institutional Review Board of the University of Kentucky College of Medicine. Human tissue samples from UPPD resulted from autopsies that were performed after obtaining informed consent using forms approved by the Institutional Review Board of the University of Pittsburgh, College of Medicine, Pittsburgh, Pennsylvania.

### Study subjects

Among the UK-ADRC cases, all autopsied subjects with detailed quantitative neuropathological data were initially considered for inclusion (*n* = 709). Cases with brain tumors or end-stage neurodegenerative diseases (Alzheimer’s disease (AD), Parkinson’s disease, dementia with Lewy bodies (DLB), prion disease, Picks disease, progressive supranuclear palsy (PSP), multiple sclerosis (MS), cerebral autosomal dominant arteriopathy with subcortical infarcts and leukoencephalopathy (CADASIL), and/or frontotemporal lobar degeneration (FTLD)) were excluded from the study. Following these exclusions, a convenience sample relatively free from brain pathologies were included (*n* = 92).

Among the UKPD cases, a set of 39 autopsied tissue samples were collected from the UKPD bio-tissue repository. The reason for using these cases was to incorporate data from younger subjects. Cases were selected by the investigators (JHN and PTN) to be free of advanced neurodegenerative pathology or any other extensive brain disease that contributed to the patient’s death. Hence, exclusion criteria included pathologically confirmed brain tumors and pathologically confirmed neurodegenerative diseases: specifically AD, DLB, and HS-Aging. The UKPD and UK-ADRC cases were combined in order to test the association between age at death and MVP density. Next, the UK-ADRC cases were used to determine the association between conventional vascular risk factors, cardiovascular/cerebrovascular diseases, neuropathological conditions, and genetic variables with MVP density. Additional cases were used from the UK-ADRC (*n* = 5) and the UPPD (*n* = 4) to include cases with severe cerebral angiopathy and chronic traumatic encephalopathy (CTE).

### Clinical and neuropathologic parameters in the UK-ADRC and UKPD data set

Clinical data were obtained from each participant’s final UK-ADRC clinical visit before death as described previously [9]. During clinical research visits, medical histories were obtained from subjects, caregivers (particularly if the subject was cognitively impaired), and/or patient records. The following self-reported vascular risk factors, cerebrovascular and cardiovascular diseases were used in the analyses: medical histories of hypertension, diabetes, hypercholesterolemia, smoking status (this is lifetime > 100 cigarettes), heart attack/myocardial infarction, congestive heart failure, stroke, traumatic brain injury, transient ischemic attack, and angina pectoralis. Responses were coded initially as unknown, absent, recent/active, or remote/inactive, and subsequently, “recent” and “remote” responses were combined into one category (i.e., history of a condition) for analytical purposes. Body mass index (BMI) values were derived from height and weight measurements and dichotomized into the following categories: < 30 BMI and ≥ 30 BMI. Age at death and sex were the only variables available on UKPD cases.

For neuropathology, brain arteriolosclerosis was operationalized as described previously, comprising a “whole-brain” score of none, mild, moderate, or severe arteriolosclerosis severity [22, 23]. Microinfarcts and total brain infarcts for each case were counted as described previously [20].

### Tissue processing and MVP calculation

To visualize MVPs, brain sections were stained with hematoxylin and eosin (H&E), alpha smooth muscle actin (α-SMA), and CD34 antibodies. α-SMA is a marker for alpha smooth muscle cells and CD34 is a marker for endothelial cells. Brain tissue processing and immunohistochemistry procedures used at the UK-ADRC have been described previously in detail [20, 24, 25]. A similar procedure was used in processing UKPD and UPPD tissue samples. Briefly, the brain was sectioned during autopsy, fixed in formalin, and processed in paraffin [20]. Afterwards, sections from archived paraffin-embedded frontal cortical were cut at 8microns thickness and placed onto glass slides for immunohistochemistry [20]. Frontal neocortex (specifically, Brodmann Area 9) was sampled, correlating with the brain area where MVPs were described previously [13]. The primary antibodies used during immunohistochemistry were α-SMA (monoclonal mouse anti-human 1A4, Dako, 1:2 dilution) and CD34 (monoclonal mouse anti-human QBEnd 10, Dako, 1:2 dilution). A biotinylated antibody (anti-mouse IgG made in horse, Vector Laboratories) was amplified using avidin–biotin substrate (ABC solution, Vector Laboratories), followed by color development in DAB (Dako). The Aperio ScanScope XT digital slide scanner was used to image the stained slide at 40X magnification creating a single high-resolution digital image [20].

For the purposes of this study, we defined an MVP as having ≥ 3 lumens within a single vascular profile. Cervos-Navarros et al. characterized vessels as having up to 10 lumens surrounded by a perivascular space [13]. Hassler et al. described MVP-type profiles as having ≥ 4 vessels running parallel to each other and surrounded by a perivascular space at a distance 10X the mean diameter of the vessels [10, 11]. We chose 3 lumens as the cutoff because a vascular profile consisting of 2 lumens on a 2-dimensional glass slide could be due to sectioning blood vessels at vascular branching points. It was previously reported that MVPs showed a tendency to occur in the frontal and parietal lobes [13] and grey matter cortical regions [10]. Our initial survey confirmed these prior reports. Therefore, MVPs were assessed within the grey matter of frontal cortex.

The CD34 primary antibody was chosen for calculating MVP density since it labels all lumens unlike the α-SMA antibody. Tissue sections were analyzed blind to the demographics, clinical and neuropathological conditions of the corresponding case. First, the entire grey matter was outlined and MVPs were counted manually using the Aperio ScanScope XT accompanying image analysis software (ImageScope) from Leica Biosystems. The formula used to calculate MVP density is

$$\frac{total\;\# \;of\;MVPs}{{grey\;matter\;area \left( {{\mu m}^{2} } \right)}}$$

### Brain tissue clearing and imaging

To visualize MVPs in three dimensions, we used a tissue clearing method called SeeDB on the case from the UK-ADRC cohort with the highest MVP density. The SeeDB method has been described previously [26]. Briefly, SeeDB involves incubating tissue samples in a series of fructose solutions of increasing concentration to match brain tissue to the refractive index of the surrounding medium. Prior to treatment with SeeDB, tissues were incubated with fluorescein-conjugated lectin (FL-1171, Vector Labs, 1:4 dilution), which labeled the endothelium of all blood vessels. Next, tissues were imaged with two-photon microscopy to capture 3-D images of the vasculature. Multiple 3-D images were stitched together using XUVtools 1.8.0 as described in Emmemlauer et al. [27], which were then viewed using Imaris 7.6.5 (Bitplane USA; Concord, MA, USA).

### Assessing glial reactivity towards single vascular profiles and MVPs

In order to assess glial reactivity in proximity to MVPs, a within-subject comparative approach was utilized involving 13 human tissue samples containing the amygdala or hippocampus, originating from the UK-ADRC cohort and featuring MVPs. The employed tissue staining process adapted the QUIVER protocol [28], albeit with a single modification. Specifically, vascular structures were visualized using the CD34 antibody (1:100, Aligent, RRID: AB_2750581) and a permanent chromogenic substrate (ImmPACT® SG Substrate Kit, Vector Cat #SK-4705). All ensuing rounds of cyclic multiplex immunohistochemistry applied the removal ImmPACT AMEC Red Substrate kit (Vector Laboratories). The staining order for subsequent rounds incorporated Ferritin (1:1000, Thermo Fisher, RRID: AB_259622), and GFAP (1:5000, Invitrogen, RRID AB_2532994), and IBA1 (1:1000, Synaptic Systems, RRID AB_2493179). A Zeiss Axio Scan Z.1 slide scanner was used after each staining round to capture complete slide images at 20 × magnification. Images were subsequently registered, deconvolved, and consolidated into a single pseudo-color image using the HALO software (Indica Labs, version 3.6).

For the quantification of glial reactivity, 20 single vascular profiles (SVPs) and 20 MVPs were randomly identified in each case, employing the CD34 channel exclusively for vascular profile identification. The outlined vascular profiles were used to establish four concentric regions at 50 μm intervals away from the vascular profile's periphery, thereby defining glial responses. The HALO software utilized two image analysis macros—the Area Quantification algorithm and the Object Colocalization algorithm—to assess the overall staining burden and the number of cellular profiles, respectively. These cellular profiles ranged in size from 50 μm to 1000 μm. The mean for the 20 for 20 SVPs and 20 MVPs were plotted as a single n for each case.

### Statistical analyses

Correlations and exploratory bivariate analyses were used to assess the association between demographics, clinical vascular risk factors, cerebrovascular and cardiovascular diseases with MVP density. For the neuropathologic variables that were ordinally scored (e.g., atherosclerosis and arteriosclerosis), non-parametric methods were used. More specifically, a Spearman’s rho correlation was used to determine the association between age at death and MVP density using UKADC (*n* = 92) and UKPD (*n* = 39) cases. Next, a Mann–Whitney U (Wilcoxon Rank Sum) test was used to determine possible risk factors for MVP pathology using only UK-ADRC cases. UKPD cases were excluded from the latter analysis due to lack of data available on clinical risk factors of interest. For testing the association between dichotomous clinical risk factors and MVP counts (as well as counts of microinfarcts and total infarcts throughout the brain), we used zero-inflated negative binomial regression (adjusting for age at death and sex), via the R statistical package (https://stats.oarc.ucla.edu/r/dae/zinb/) [29]. Other statistical analyses were performed using IBM SPSS Statistics 22 Properties and PC-SAS 9.34 (SAS Institute, Inc.; Cary, NC, USA).

## Results

**Fig. 1:**
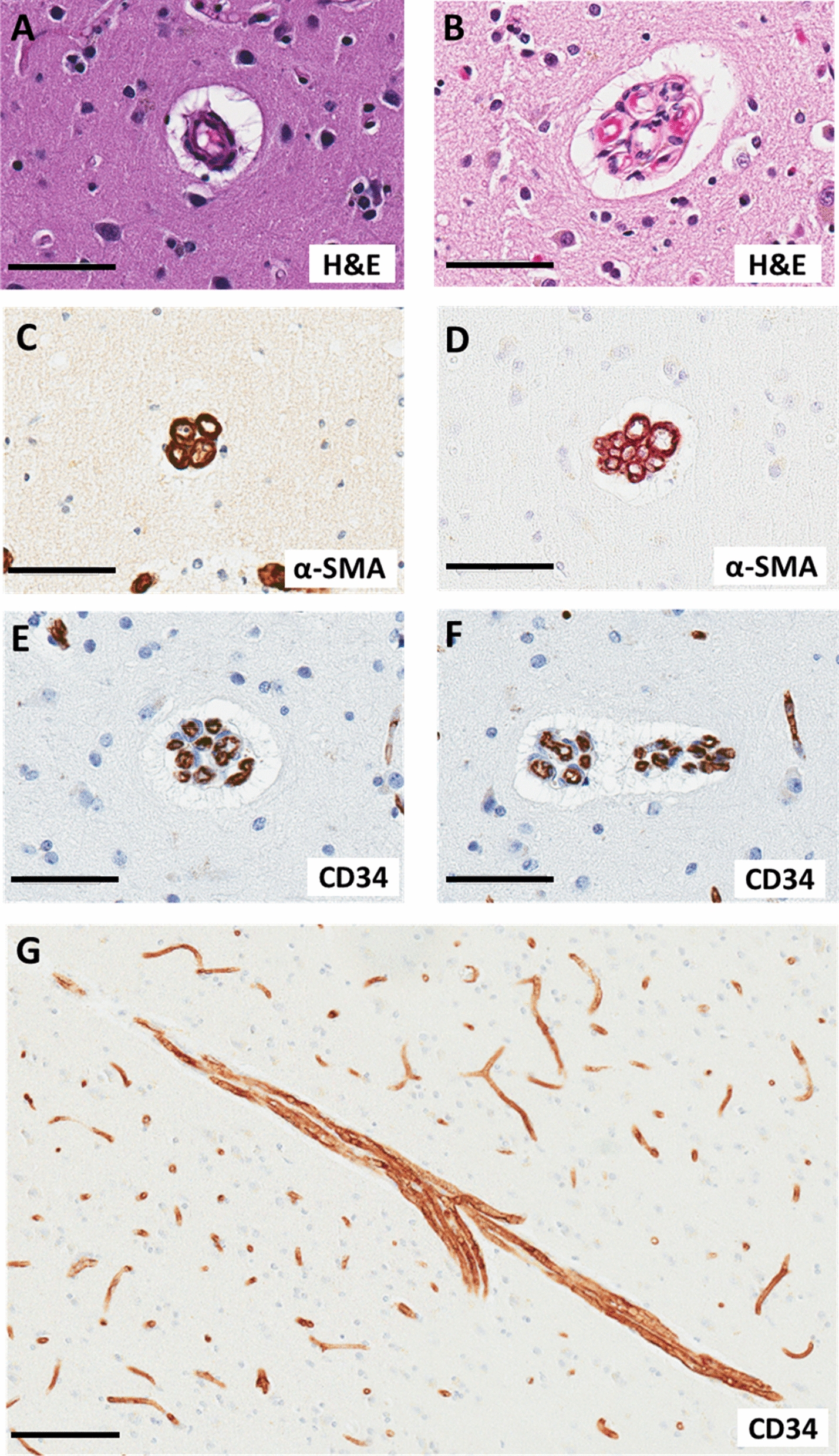
Multi-lumen vascular profiles (MVPs). MVPs are vascular beds consisting of ≥ 3 lumens enclosed in a perivascular space on a cross-sectional view. **A**, **B** Photomicrographs of hematoxylin-and-eosin stained blood vessels within the grey matter of frontal tissues. **A** What we assume to be a normal arteriole (due to its structure and size) from a 42 year-old female. **B** What we describe as a MVP with at least 3, lumens, some of which contain red blood cells, from a 96 year-old female. **C**, **D** Photomicrographs of alpha smooth muscle actin (α-SMA) stained MVPs in cross-section. **C** shows an MVP with at least 4 lumens of similar size from a 91 year-old female. **D** shows a MVP with at least 13 lumens of varying size from a 89 year-old male. **E**–**G**) Photomicrographs of CD34 stained MVPs. **E**, **F** show MVPs with at least 10 lumens in cross-section from a 89 year-old male. **G** shows a MVP cut in a longitudinal direction from a 91 year-old female. Scale bars: a-f = 50 µm, g = 100 µm

Some characteristics of the study sample are shown in Table 1 and Additional file 1. Included individuals from the UK-ADRC cohort were predominantly white race (data not shown). Race/ethnicity information was not available for cases within the UKPD brain repository. The overall median age at death for cases used in the study was 84.0 years with an interquartile range of 34.0 years (Table 1). The overall percentages of females and males within this study was 52.7% and 47.3% respectively (Table 1). In order to convey examples of vascular profiles representing the spectrum of MVP pathology, images were obtained from human brain sections stained with H&E, α-SMA, or CD34 (Fig. 1).

**Table 1:** Demographics of included cases from the University of Kentucky Pathology Department (UKPD) and University of Kentucky Alzheimer's Disease Research Center (UK-ADRC) cohorts*

	UKPD cohort	UK-ADRC cohort	Overall
Sample size	39	92	131
Demographic variables			
Age at death, median (IQR)	38.0 (18.0)	87.0 (11.0)	84.0 (34.0)
Sex (%)			
Male	59.0	42.2	47.3
Female	41.0	57.6	52.7

*Cases from both cohorts were combined in order to test the association between age at death and MVP density

**Fig. 2:**
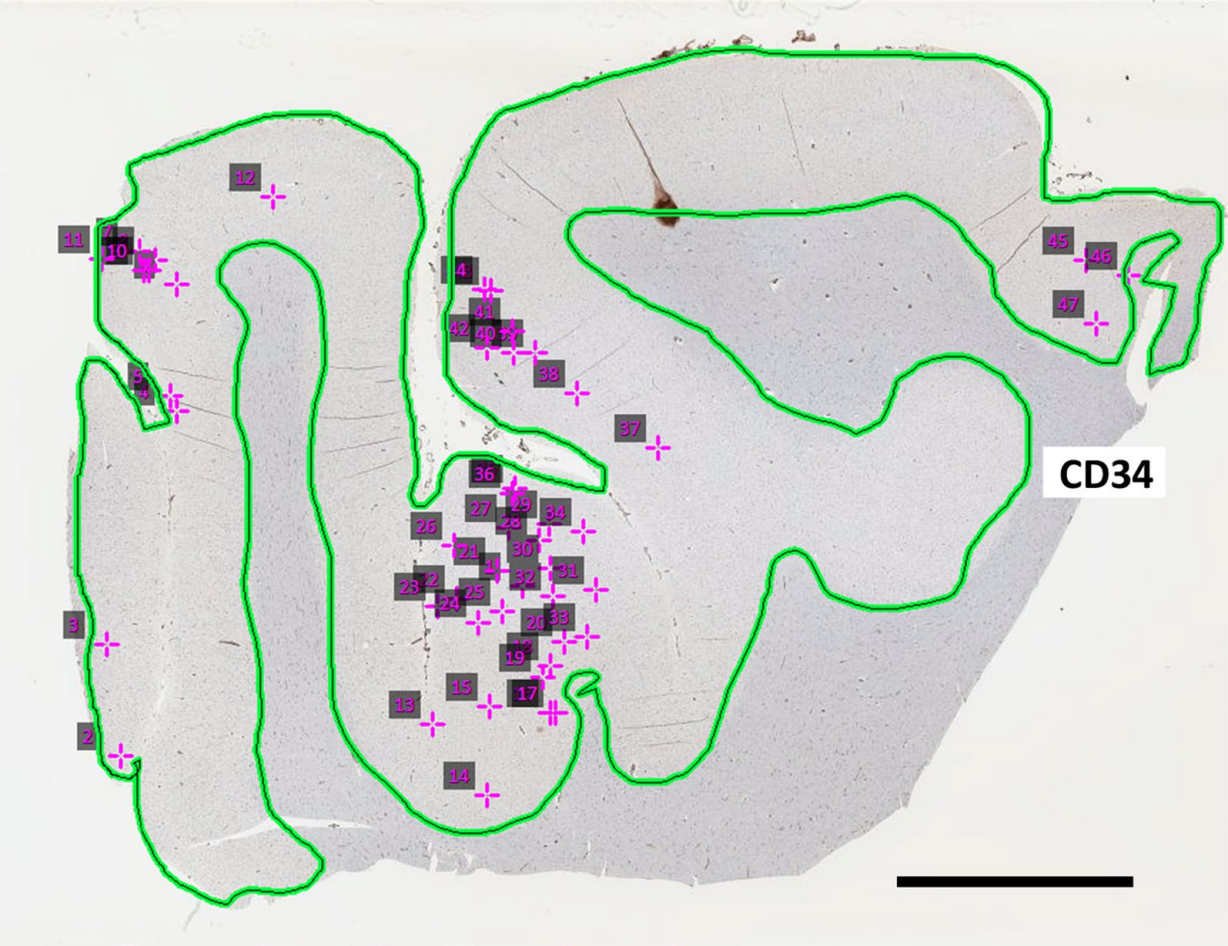
Schematic of MVP quantification. The photograph is of a CD34 stained tissue from the frontal cortex (Broadmann area 9) of an 84 year-old female. Using Aperio ScanScope digital slide scanner accompanying image analysis software, the grey matter area was outlined (green-black line). Next, the grey matter area was manually scanned for MVPs which were marked by a counter (pink crosses). The total # of MVPs and the grey matter area for each case were used to calculate an MVP density. Scale bar = 5 mm

**Fig. 3:**
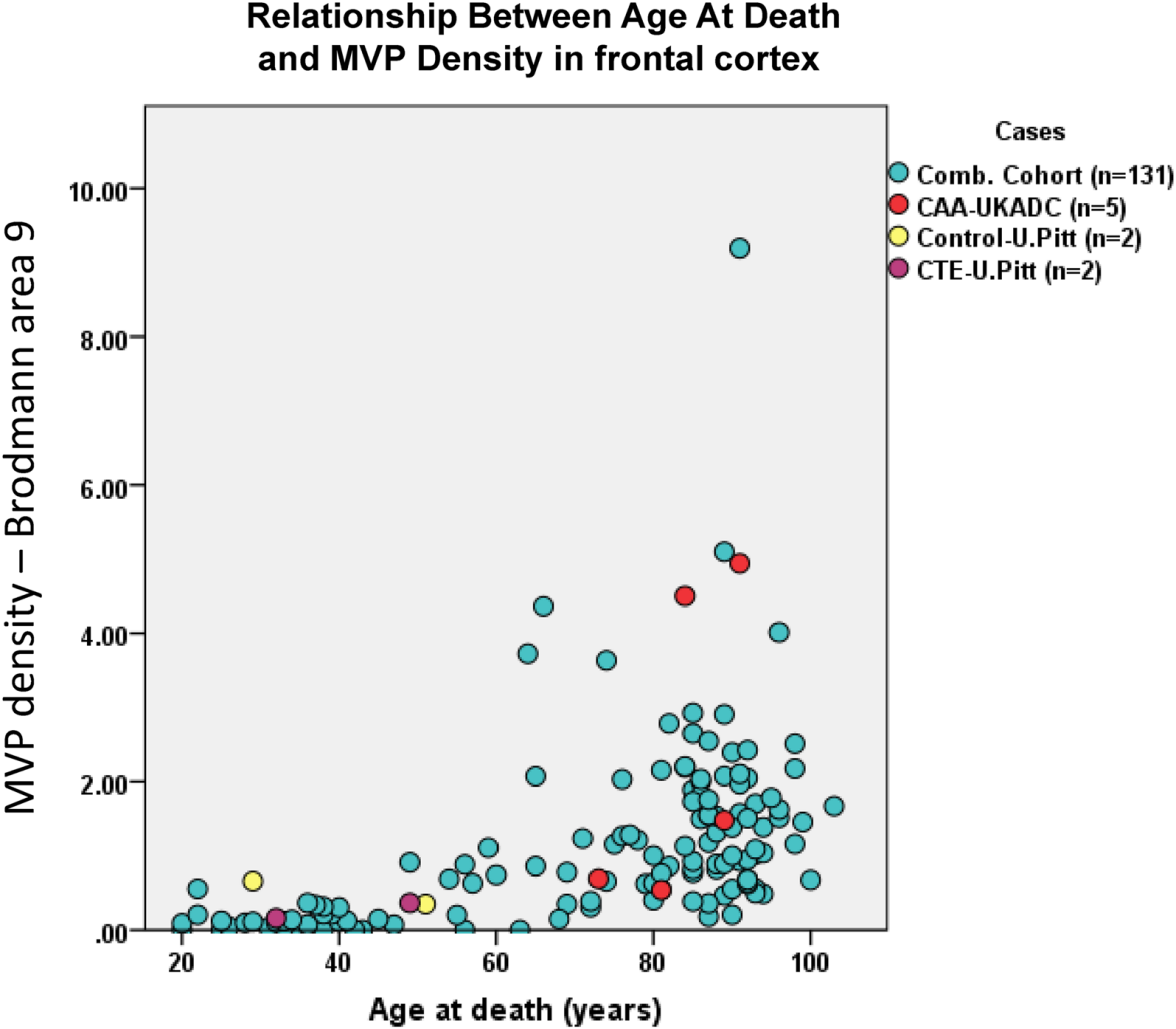
Relationship between age at death and MVP density (counts per 10^7^ microns^2^). Cases from the UKPD and the UK-ADRC cohort (blue dots) were combined in order to determine the association between age at death and MVP density. A scatter plot was used to show each case’s MVP density with corresponding age at death. Using a Spearman’s rho test, the correlation between age at death and MVP density was 0.60 with a p-value of < 0.0001. Cases with severe CAA (red dots) from the UK-ADRC and 2 with CTE (purple dots) and aged-matched controls (yellow dots) were also included. *Comb*. combined, *U*. university, *CAA* cerebral amyloid angiopathy, *CTE* chronic traumatic encephalopathy, *MVP* multi-lumen vascular profiles

The quantification method of MVPs in this study is depicted in Fig. 2. Using all 131 cases, there was a significant linear correlation between age at death and MVP density as shown in Fig. 3 (Spearman’s rho = 0.60; *p* < 0.0001). In other words, age at death was associated with MVP density. Brain tissue samples from the UK-ADRC (*n* = 5) and UPPD (*n* = 4) were used in two separate preliminary studies in order to observe the relationship between cerebral amyloid angiopathy (CAA) and chronic traumatic encephalopathy (CTE) with MVP density. There were no detectable visible relationship between CAA or CTE with MVP density (Fig. 3) in these relatively small samples.

Among UK-ADRC cases (*n* = 92) with clinical, neuropathological, and genetic information, the majority of cases had hypertension (59.0%), hypercholesterolemia (58.4%), and the *APOE* ɛ3/ɛ3 genotype (64.6%) (Additional file 1). In order to determine the clinical vascular risk factors for MVP pathology, a Wilcoxon rank-sum test was performed on 17 clinical variables. None of the clinical variables were significantly associated with MVP density within this cohort, following correction for multiple comparisons. However, a nominal association was noted between a clinical history (self-reported) of brain trauma and MVPs (Table 2). Otherwise, there were no significant associations between MVPs in frontal cortex and demographic (sex), neuropathological (B-ASC and atherosclerosis), or genetic (*APOE*) variables (Table 3).

**Table 2:** Prevalence ratios (PR) with 95% CI* between dichotomous clinical variables and lesion counts of frontal cortex MVPs, microinfarcts, and total brain infarcts

Clinical history (mostly by self-report)	MVPs in frontal cortex		Microinfarcts		Total brain infarcts	
	PR	95% CI	PR	95% CI	PR	95% CI
Myocardial infarction	1.58	(0.745, 3.33)	0.98	(0.48, 2.009)	1.34	(0.595, 3.038)
Chronic heart failure	0.89	(0.449, 1.765)	1.42	(0.705, 2.85)	0.98	(0.431, 2.237)
Hypertension	1.91	(0.908, 4.026)	0.67	(0.232, 1.937)	1.91	(0.673, 5.437)
Stroke	0.75	(0.355, 1.592)	**3.88**	**(1.824, 8.249)**	**3.19**	**(1.607, 6.34)**
Transient ischemic attack (TIA)	0.81	(0.304, 2.176)	1.16	(0.451, 3)	1.69	(0.706, 4.051)
Brain trauma	**2.07**	**(1.094, 3.924)**	1.22	(0.545, 2.725)	2.19	(0.938, 5.123)
Diabetes	1.25	(0.608, 2.582)	**2.50**	**(1.272, 4.903)**	**2.87**	**(1.414, 5.812)**
Angina pectoralis	1.04	(0.485, 2.249)	0.54	(0.184, 1.574)	1.03	(0.499, 2.107)
Peripheral vascular disease	1.66	(0.842, 3.271)	1.97	(0.888, 4.377)	1.55	(0.622, 3.859)
Coronary artery bypass graft	2.48	(0.802, 7.687)	1.41	(0.476, 4.173)	1.06	(0.337, 3.332)
Smoking	1.52	(0.747, 3.093)	1.03	(0.353, 2.987)	0.62	(0.276, 1.401)

*Zero-inflated negative binomial regression was performed including age at death and sex in the model. Nominal confidence intervals (95% CI) are shown, which are unadjusted for multiple comparisons. Bold: nominally statistically significant at *p* < 0.05

**Table 3:** MVP densities: test for association with ordinal clinical, neuropathological and genetic (*APOE*) variables

	MVP Density (#/µm)	Statistical significance
Demographics, median density (IQR)		
Sex		
Male	1.18 (1.34)	NS
Female	1.38 (1.31)	
Neuropathological variables		
Atherosclerosis		NS
None	0.82 (0.42)	
Mild	1.63 (1.62)	
Moderate	1.39 (1.21)	
Severe	1.36 (1.29)	
Almost occluded	0.97 (0.85)	
B-ASC		NS
None	0.92 (0.92)	
Mild	1.54 (1.17)	
Moderate	1.31 (1.60)	
Severe	0.80 (NA)	
Genetic variables		
* APOE* genotype		NS
ℇ2/ℇ3	1.30 (1.73)	
ℇ3/ℇ3	1.32 (1.01)	
ℇ3/ℇ4	1.19 (1.62)	

*A Mann–Whitney U (Wilcoxon Rank Sum) test was applied. To correct for multiple comparisons, an adjusted significance level of 0.00002 (17 comparisons) was used to determine significance

*IQR* interquartile range; *NS* not significant at *P* < 0.05; *MVP* multi-lumen vascular profiles; *B-ASC* brain arteriolosclerosis as defined previously [22, 23]

**Fig. 4:**
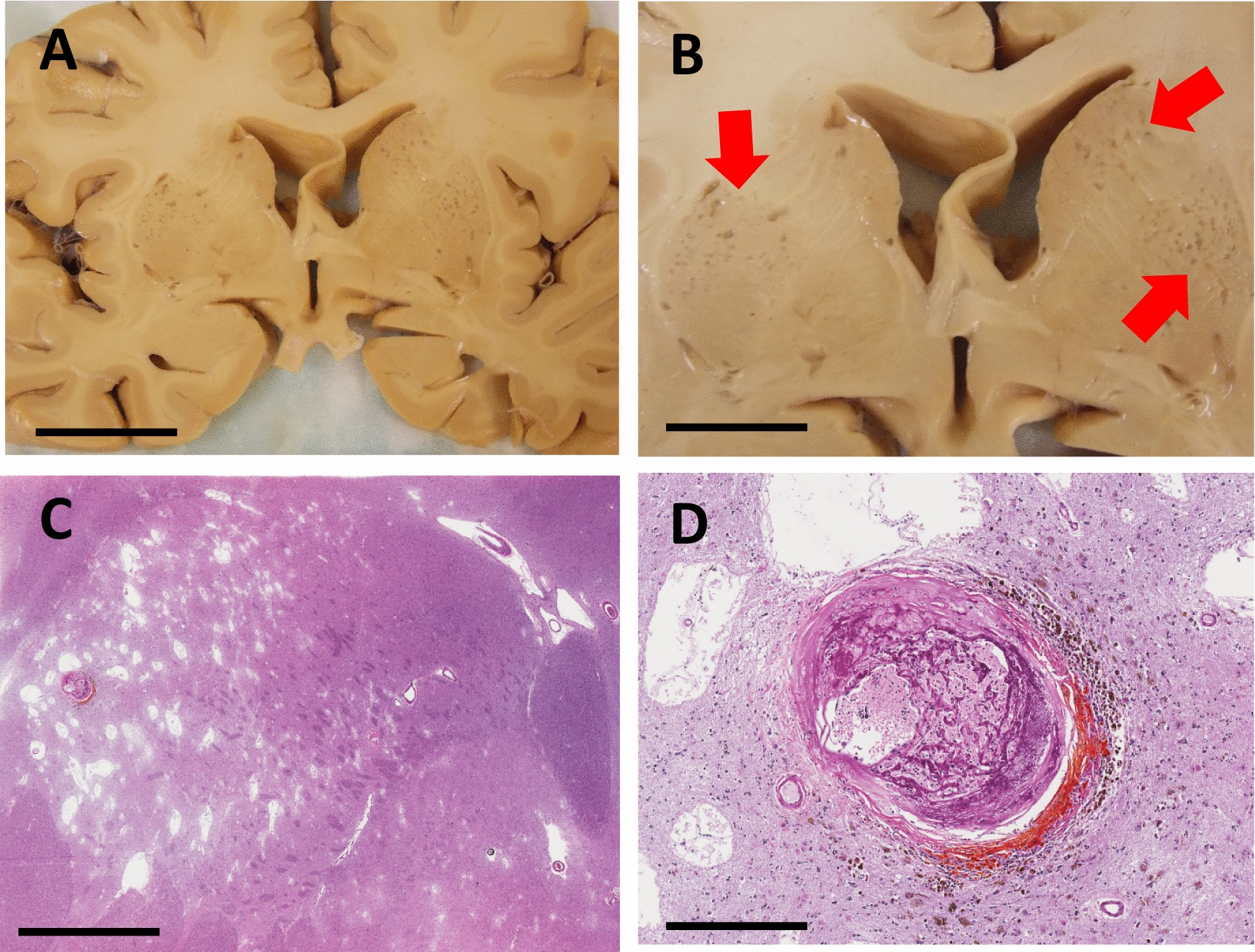
UK-ADRC case with highest MVP density. **A**, **B** Photographs showing the gross anatomy of the basal ganglia from a 89 year-old male. **B** Inset of (**A**) with red arrows indicate holes in the basal ganglia. **C**, **D** Photomicrographs of hematoxylin-and-eosin stained tissue sections from the putamen of a 89 year-old male. **C** shows regions of infarct tissue. **D** shows what we presume to be a cerebral microaneurysm (Charcot-Bouchard aneurysm). Scale bars: a = 2 mm, b = 2 cm, c = 4 mm, d = 500 µm

**Fig. 5:**
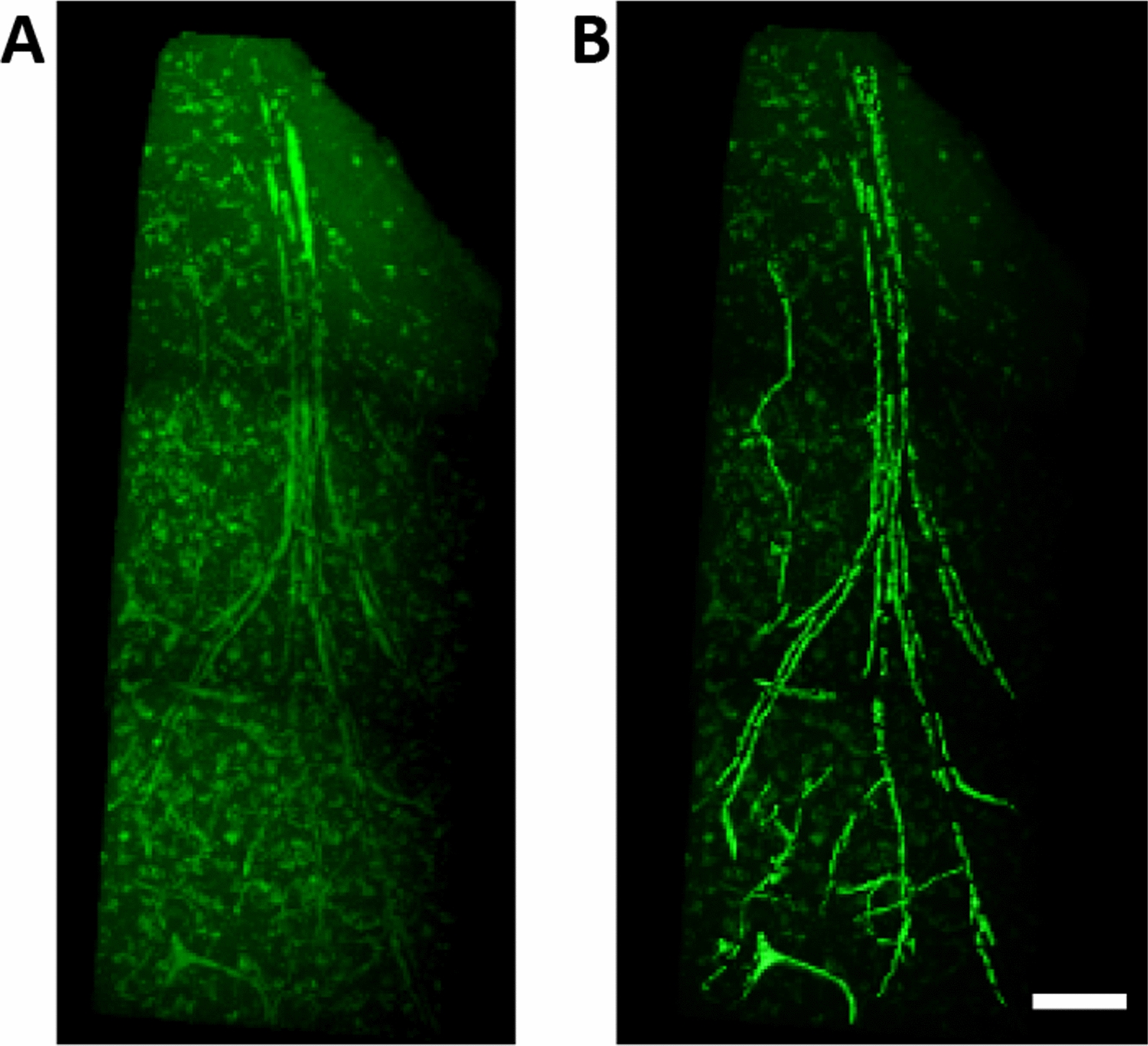
3-D Visualization of MVPs using SeeDB Method. Photomicrographs show the 3D branching of MVPs within the frontal cortex of a 89 year-old male. Panel **A** shows the raw data, and the image in panel **B** was surface-rendered and isolated from other non-vascular objects using Imaris for improved visibility of the MVPs. Scale bar = 100 mm

Further analyses were performed on the UK-ADRC case with the highest MVPs of the sample group, which was an 89 year-old male with a clinical history of hypertension. The gross anatomy of his brain showed dilation of perivascular spaces (**état criblé)** in the basal ganglia (Fig. 4). A microhemorrhagic region with distortion of the vessel wall and perivascular reaction (could be designated a microaneurysm) was seen in the patient’s putamen (Fig. 4 C, D). Using an optical tissue clearing method combined with multi-photon imaging, the 3D visualization of this patient’s MVPs showed extensive vascular lumens in multiple Virchow-Robin spaces. Shown in Fig. 5 is a 2-D rendering of a 3-D image of a single large MVP branching into at least 4 smaller MVPs.

**Fig. 6:**
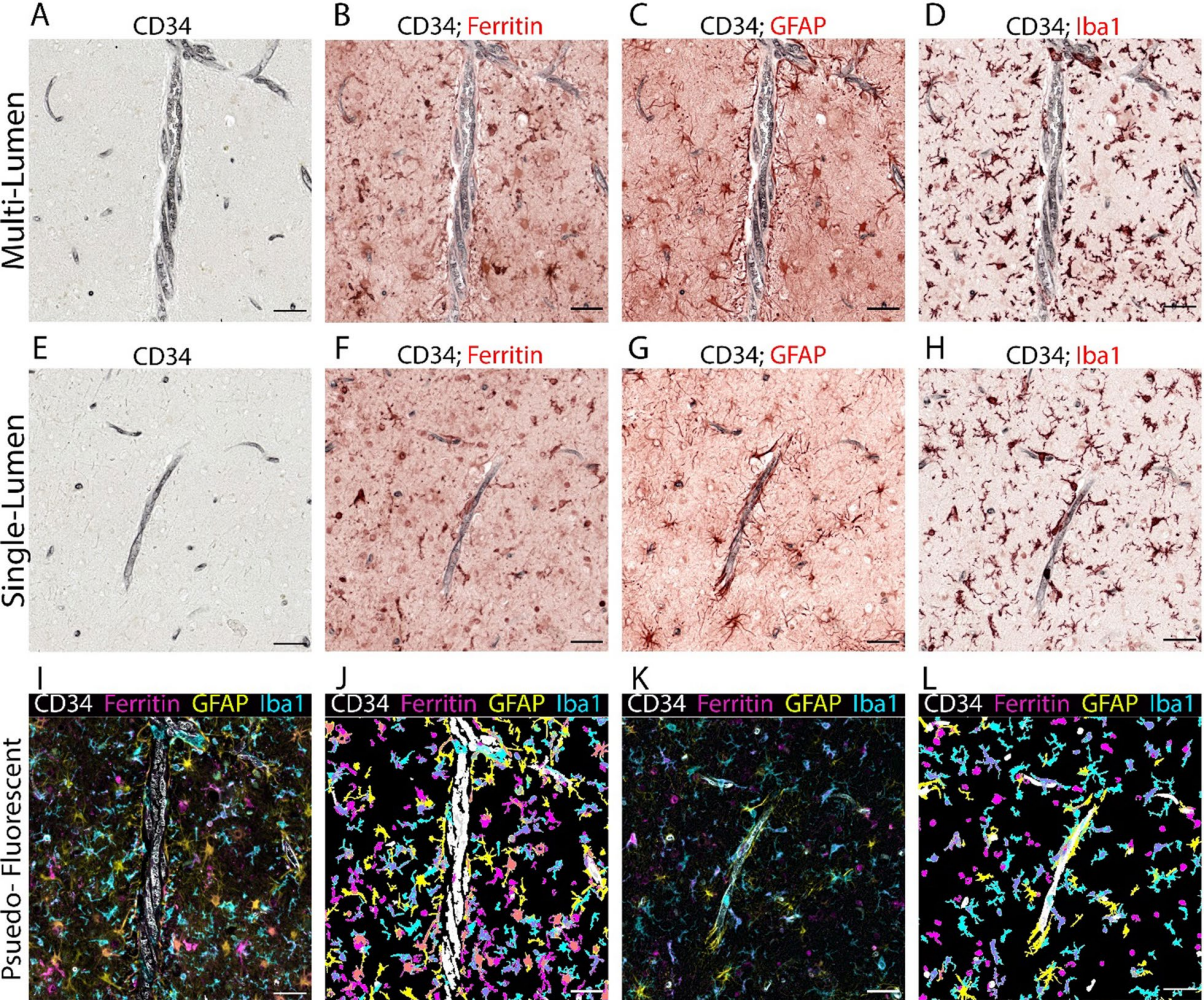
Multiplexed staining of glia in relation to vascular profiles and analysis of Vascular Phenotypes A sequential multiplexed staining and analysis, known as QUIVER, was employed on human FFPE tissue. The procedure started with the staining for CD34 to visualize multiple vascular profiles (MVP) and single vascular profiles (SVP) in the same case (**A**). This step utilized a permanent chromogen to preserve the staining throughout each subsequent round. Subsequent staining rounds were performed for ferritin (**B**, **F**), GFAP (**C**, **G**), and IBA1 (**D**, **H**), sequentially, using a removable chromogen. Post-deconvolution of single-channel IHC images, merged pseudo-fluorescent images were generated for MVP (**I**, **J**) and SVP (**K**, **L**). Cell count data was produced using the object colocalization algorithm in the HALO software, and digital markup (**J** and **L**). The color labels correspond with the representative color in the markup image and merged image. Scale bars = 50 μm

**Fig. 7:**
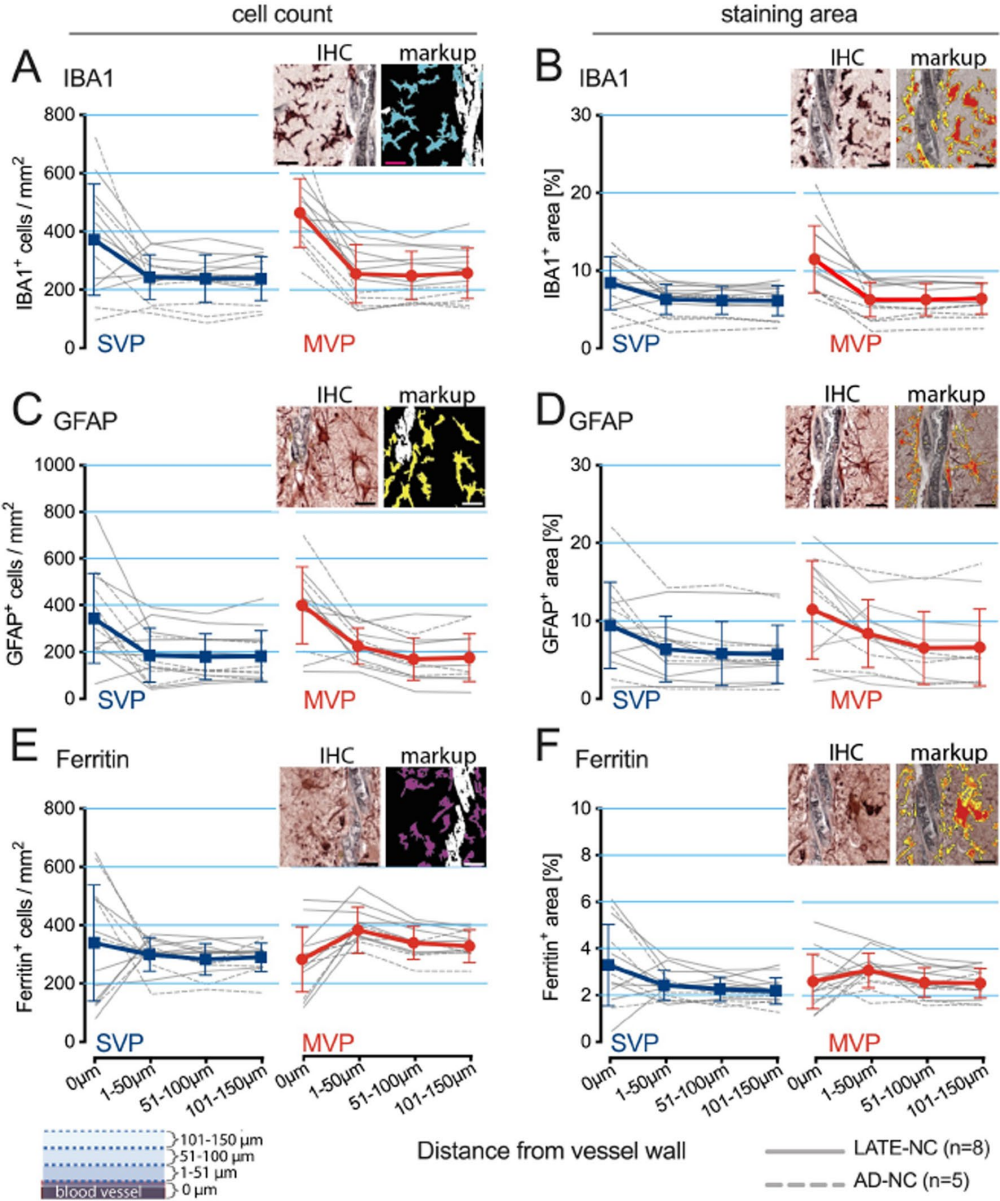
Digital analysis of glial populations near vascular structures. To explore the glial populations' relationship with vascular structures, a digital analysis method was used. First, we identified each vascular structure using CD34 staining. Next, the vascular space outline was created, forming a digital concentric layer at 50 μm intervals. With Halo software, we generated automated tissue analysis markups for cell quantification via the object colocalization algorithm and staining area determination through the area fractionator algorithm, as the inset illustrates. We obtained cell counts and area of positive staining for IBA1 (**A**, **B**), GFAP (**C**, **D**), and ferritin (**E**, **F**), respectively. The gray lines are mean values for each person. With the dashed lines for the ADNC cases and the solid lines for the LATE-NC cases. The color lines show the mean ± SD for call the cases. Analysis of the overall staining area revealed similar trends to the cell counts, with the most striking difference noted between MVP and SVP in the IBA1-positive cells associated with the blood vessel. The results presented are the mean per case for 20 MVPs and 20 SVPs quantified each time. Scale bars = 25 μm

In our next phase of analysis, we explored the potential presence of increased glia reactivity in association with MVPs compared to SVPs. This was accomplished by using a marker for microglia (IBA1), astrocytes (GFAP), and glia that are presumably reactive to iron (ferritin). Upon visual inspection, we observed an evident increase in glia in proximity to certain MVPs, when compared to an SVP of similar size (Fig. 6). An average of 20 profiles per each of the 13 cases was drawn upon for quantification of the staining, which demonstrated that the extent of glia reactivity within a 200 μm radius from the vascular profile was similar between the SVPs and MVPs (Fig. 7).

## Discussion

We studied MVPs in cases from the UK-ADRC, UKPD, and UPPD brain repositories. In this sample, age at death was associated with MVP density. When analyzing the association between conventional vascular risk factors (e.g., hypertension, diabetes), cardiovascular diseases (e.g., heart attack, arrhythmia), and cerebrovascular disease (e.g., stroke, transient ischemic attack), we did not find an association between these variables and MVP density. Further, when analyzing the association between neuropathological (e.g., brain arteriolosclerosis) and genetic (e.g., *APOE* genotype) variables of interests, we also did not find association between these variables and MVP density. Since before 1900, evidence has been gathered about a number of changes that can affect the cerebral micro-vasculature in aging human brains, e.g. aneurysms, fistulas, ectatic walls, tortuosity, collagenous changes, and others; the complexity of the terminology has mirrored that of the histological observations (Table 4). In the present study, our goal was to provide impetus for the study of MVPs in particular, which are a common correlate of brain aging.

**Table 4:** Terminology used to refer to human cerebral multi-lumen microvessel

References	Term for human cerebral multi-lumen vessel	Definition applied
(*Present study*)	Multi-lumen vascular profiles (MVP)	≥ 3 adjacent vascular lumens enclosed in a perivascular space
[17–19]	Raspberries	≥ 3 adjacent vascular lumens, varying in size from 20 to 80 μm
[16, 36, 38]	Vascular convolutes or convoluted vessels (most frequently used prior term[s], overlapping with, but not exactly corresponding to, MVPs)	Not formally defined, but depicted in various published papers in histological, ultrastructural and angiogram preparations. Some of these terms were applied to vessel beds that did not have multiple lumens
[10, 11, 13–16, 18, 19, 39]	Additional related terms: Vascular loops, vascular bundles, vascular multiplications, vascular glomerular loop formations, vascular wickerworks, "extreme tortuosity, [and] multiplications … of the smallest arterioles and lumpy-bumpy capillaries" (these terms were used in different combinations in various studies, and did not necessarily overlap exactly with MVPs)	

There are some limitations in this study [9]. The UKPD, UPPD, and UK-ADRC brain repositories are vulnerable to selection bias and information bias [21]. The UKPD brain bank is a collection of tissue samples from persons that come to autopsy from a tertiary-care hospital, which carries biases associated with hospital affiliated autopsy services. With respect to UKPD cases, some clinical, neuropathological, and genetic information of interest were not available for this study. Therefore, we could not assess MVP risk factors within a younger cohort. The UK-ADRC brain repository is a community and clinically based cohort of research subjects associated with an ADC, which carries other known biases [21, 30–33]. As a result, UK-ADRC participants are predominantly white race, highly educated, and at risk for developing clinical AD [21, 31, 34]. Due to the lack of socioeconomic information and low sampling of individuals from different racial/ethnic groups, race and ethnicity were not included in the analyses. The data on clinical disease risk factors are largely self-reported, which can lead to an underestimation of the true disease frequencies [35]. In addition, duration of disease (e.g. hypertension, diabetes) data was not available.

Despite the challenges inherent to a retrospective cross-sectional study, the combined cohort we used for our study provided brain tissue samples that span a broad aging spectrum (up to >100 years at death). In addition, the UK-ADRC provides detailed clinical, neuropathological, and some genetic information to study correlations. The information allowed us to test the associations between conventional vascular risk factors (e.g. hypertension, diabetes), cardiovascular/cerebrovascular diseases (e.g., heart attack, stroke), and neuropathological diseases (e.g., B-ASC, atherosclerosis) with MVPs.

In this autopsy sample, we showed that MVP density is associated with age at death. Prior studies have provided conflicting evidence for the association between age at death and the presence MVP-type pathologic features. In a study of 231 cases (age of death range: 1–90 + years), Hassler reported that MVP-type structures were not seen in cases with an age at death of ≤ 39 years [11]. Within the same study, Hassler recorded MVP-type structures in cases with an age of death ≥ 48 years [11]. In a study of 8 cases (age of death range: 26–88 years), Cervos-Navarro et al., showed that all the aged patients (61–88 years) had cerebral MVP-type structures [13]. However, no MVP-type structures were found in the young cases (26–48 years) within the same study [13]. In a study of 70 cases (age of death range: 36–91 years), Arsene et al. observed that MVP-type structures were only present in 2 cases with an age at death of 56 and 80 years respectively [14]. The varying results could be attributed to differences in tissue sampling, staining techniques, MVP characterization/quantification, and cause of death. Instead of dichotomizing the presence of MVPs, we calculated a density score in order to account for grey matter area variability. We also used immunohistochemical methods which allowed for enhanced visualization of blood vessels.

In the present study, it was difficult to determine conclusively the types of blood vessel(s) (i.e., small arteries, arterioles, capillaries, venules, and/or small veins) that were mostly affected within the aged brain. Moreover, there is not a consensus within the literature as to which vessels are affected [13]. We certainly found MVPs that positively stained for both α-SMA (smooth muscle marker) and CD34 (endothelium) indicating that MVP profiles often affect arterioles. Using electron microscopy, Cervos-Navarro et al. found that the lumen within each MVP-type structures had a continuous endothelial cell layer with tight junctions [13]. In addition, the basement membrane of the endothelial cell layer and smooth muscle cell layer formed a homogenous layer within the MVP-type structures [13]. With these findings, the authors concluded that MVP-type structures are an arteriolar phenomenon [13] which is consistent with our findings, although we did find more MVPs using the CD34 marker which indicates that some capillaries may be MVPs.

The biological implications of brain MVPs are not fully understood. It could be part of normal aging, a pathological condition, or a compensatory mechanism. This has been a long-standing scientific puzzle. As stated by Rudolf Altschul in 1944, the “c.v. [convoluted vessels] have been variously interpreted. Carletti and others have considered them as pathological alterations; Pfiefer considered them to be a physiological device. My own view is that they are common malformations.” [36] (with reference to [16, 37]). The appearance of MVPs could be affected by shrinking of surrounding parenchymal tissue with subsequent vessel distortion [10, 14, 15] or some other agonal or tissue fixation artifact [15], but their presence is not entirely an artifact of tissue processing in our opinion. Other authors have discussed that MVP development could be due to vessel recanalization [13], a result of increased secretion of angiogenic factors in response to chronic ischemia leading to vessel proliferation and/or elongation [13–15], or modified activity of local matrix metalloproteinases [14]. Our study showed minimal glia reactivity around MVPs, which lends support to the assertion that MVPs are not catalysts for gliosis and indicating that these changes are not necessarily linked with acute vascular injury in the vicinity of these vessels. Future directions may also include correlating MVPs with angiogenesis factors. More experiments are needed in order to fully understand the development mechanism of MVPs in the brain and how their morphological differences might impact distribution of blood to the capillary bed.

There is little information published on the risk factors and co-pathologies of MVPs in the brain. In a study of 231 cases (age of death range: 1–90 + years), Hassler described that a greater proportion of men had MVP-type structures compared to females in that sample [11]. In addition, the author reported that heart weights and arteriosclerosis severity were higher in cases with MVP-type structures [11]. However, statistical analyses were not reported. In our study, we tested the association between conventional vascular risk factors, cardiovascular diseases, and cerebrovascular diseases/pathologies with MVP density. We did not find a statistically significant association between the variables we tested and MVP density. However, a larger sample size or more focused hypothesis-testing may prove that our sample size was statistically underpowered to identify a true association, should it exist. The marginal association found in the current study between a clinical history of brain trauma and MVPs in frontal cortex is intriguing. By contrast, other types of cerebrovascular pathologies (microinfarcts or total infarcts) were not associated with brain trauma. Given the sample size and related statistical considerations, this suggestive finding will require further follow-up.

An important set of observations relevant to MVPs were made previously in three papers from Ek Oloffson et al. [17–19], reporting on brain autopsy findings in a hospital-based cohort from Lund, Sweden. In these papers, the authors reported vascular structures that were operationally defined the same as MVPs and termed them “raspberries” (Table 4). In the most recent paper on raspberries by Ek Oloffson et al. [19], the cohort comprised a total of 141 subjects. A trend for association was found between the vascular raspberries and atherosclerosis. There were no other associations noted in that sample. We note that despite the common operationalization with MVPs, the term “raspberries” (and most of the photomicrographs in the Ek Oloffson papers [17–19]) imply a bulbous multi-lumen tip of a vessel, analogous perhaps to a kidney glomerulus, or alternatively a cluster-type arrangement, whereas our findings underscore that the multi-lumen vascular architecture often continues for considerable length along the longitudinal axis of cerebral vessels.

In conclusion, MVPs are an aged-related brain pathology whose risk factors may relate to brain trauma and may not include conventional vascular risks factors. In our study, MVPs were not associated with sex, cardiovascular diseases, or cerebrovascular diseases/pathologies. As investigators have found evidence of these intriguing structures across the world, we hope to help harmonize these studies to better understand the biological mechanisms of cerebrovascular function, plasticity, and degenerative changes.

### Supplementary Information

**Additional file 1** Supplemental Material.
