# Supplementary material for: A neuropathologic feature of brain aging: multi-lumen vascular profiles
Source: Acta Neuropathol Commun. 2023 Aug 28;11:138. doi: 10.1186/s40478-023-01638-2 (PMC10464008; doi:10.1186/s40478-023-01638-2)
Supplement: Supplementary file 1 — Additional file 1 Supplemental Material. [file 40478_2023_1638_MOESM1_ESM.docx]

**Supplementary Table**

**Supplementary Table 1**: Clinical, Neuropathological, and Genetic Information of UK-ADRC cases used in MVP study. Clinical variables were self-reported and neuropathological variables were semi-quantitatively graded. There were not individuals with the ApoE ɛ4/ɛ4 genotype in our study sample. These variables were used to determine the risk factors for MVP density. The number of cases missing information on a given variable is reported. Clinical, neuropathological, and genetic information on cases from the UKPD were not available.
